# *Epimedium koreanum* Nakai Displays Broad Spectrum of Antiviral Activity *in Vitro* and *in Vivo* by Inducing Cellular Antiviral State

**DOI:** 10.3390/v7010352

**Published:** 2015-01-20

**Authors:** Won-Kyung Cho, Prasanna Weeratunga, Byeong-Hoon Lee, Jun-Seol Park, Chul-Joong Kim, Jin Yeul Ma, Jong-Soo Lee

**Affiliations:** 1 Korean Medicine (KM) Based Herbal Drug Development Group, Korea Institute of Oriental Medicine, Deajeon 305-764, Korea; E-Mail: wkcho@kiom.re.kr (W.-K.C.); 2 College of Veterinary Medicine, Chungnam National University, 220 Gung-Dong, Yuseong-Gu, Daejeon 305-764, Korea; E-Mails: prasannapdn05@gmail.com (P.W.); byeonghoon_2@naver.com (B.-H.L.); pjs123a@naver.com (J.-S.P.); cjkim@cnu.ac.kr (C.J.K.)

**Keywords:** *Epimedium koreanum* Nakai, herbal medicine, quercetin, antiviral effect, anti-influenza Effect

## Abstract

*Epimedium koreanum* Nakai has been extensively used in traditional Korean and Chinese medicine to treat a variety of diseases. Despite the plant’s known immune modulatory potential and chemical make-up, scientific information on its antiviral properties and mode of action have not been completely investigated. In this study, the broad antiviral spectrum and mode of action of an aqueous extract from *Epimedium koreanum* Nakai was evaluated *in vitro,* and moreover, the protective effect against divergent influenza A subtypes was determined in BALB/c mice. An effective dose of *Epimedium koreanum* Nakaimarkedly reduced the replication of Influenza A Virus (PR8), Vesicular Stomatitis Virus (VSV), Herpes Simplex Virus (HSV) and Newcastle Disease Virus (NDV) in RAW264.7 and HEK293T cells*.* Mechanically, we found that an aqueous extract from *Epimedium koreanum* Nakai induced the secretion of type I IFN and pro-inflammatory cytokines and the subsequent stimulation of the antiviral state in cells. Among various components present in the extract, quercetin was confirmed to have striking antiviral properties. The oral administration of *Epimedium koreanum* Nakai exhibited preventive effects on BALB/c mice against lethal doses of highly pathogenic influenza A subtypes (H1N1, H5N2, H7N3 and H9N2). Therefore, an extract of *Epimedium koreanum* Nakai and its components play roles as immunomodulators in the innate immune response, and may be potential candidates for prophylactic or therapeutic treatments against diverse viruses in animal and humans.

## 1. Introduction

Many viral infections pose a great danger to humans and livestock, often causing deaths and significant economic losses. For instance, influenza spreads around the world in seasonal epidemics, resulting in approximately three to five million yearly cases of severe illness and approximately 250,000 to 500,000 yearly deaths [1]. During the previous century, deadly viruses have caused pandemics worldwide on a number of occasions [2]. Moreover, new and re-emerging infectious viral diseases will pose a rising global health threat, and the risk of spreading these viruses between continents and countries is even larger [3]. HIV/AIDS, Severe Acute Respiratory Syndrome (SARS), and the recent 2009 pandemic H1N1 influenza are only a few of many examples of emerging infectious diseases in the modern world [4].

A number of preventative and therapeutic measures, including biosecurity, vaccination and antiviral drugs, are routinely used to prevent and treat viral diseases. Vaccines form the basis for the prevention of many viral infections, but there are substantial drawbacks [5]. For the influenza virus only, vaccination failures have been widely documented, and in the elderly population, in which most of the mortality occurs, vaccines are only approximately 50% effective [6]. Moreover, in the eventuality of a pandemic infection with a new strain, antiviral drugs represent the first line of defense [7]. Due to their metabolic properties, viruses are difficult to control, and there are relatively few drugs for the treatment of viral diseases. However, the side effects associated with the central nervous system and the gastrointestinal tract, as well as the rapid emergence of antiviral resistance during therapy, has limited the usefulness of these drugs [8,9]. Therefore, innovative strategies and responses are required to come across the economic and human health risks associated with viral diseases.

Historically, natural herbal medicines have been used by many societies for the treatment of human diseases. Approximately 20,000 plant species used for medicinal purposes are reported by the WHO [10]. In particular, a lot of extracts or substances from medical herbs or plants have been reported to have antiviral effects against infectious viruses [11]. Therefore, extracts or natural products, as pure compounds or standardized plant extracts, provide unlimited opportunities for new antiviral drugs with high efficacy, low toxicity and minor side effects.

*Epimedium koreanum* Nakai is an evergreen, perennial flowering plant that belongs to Family Berberidaceae, and the aerial parts of the plant have been widely used in traditional Korean and Chinese herbal medicine to treat infertility, impotence, neurasthenia, cardiovascular diseases, amnesia, lumbago, arthritis, various immune-modulatory problems, and also as an aphrodisiac, and anti-rheumatic, for thousands of years [12,13,14]. Additionally, recent pharmacological studies demonstrate that *Epimedium koreanum* Nakai contains anti-inflammatory, hypotensive, anti-oxidant and anti-tumor activities [15,16,17,18]. Importantly, it has been shown that *Epimedium koreanum* Nakai enhances the immune function [19,20]. However, the antiviral effect of *Epimedium koreanum* Nakai has not been completely investigated. Moreover, despite having multiple biological properties, available scientific data on *Epimedium koreanum* Nakai’s immune-modulatory potential and responsible molecules have not been reported.

In this study, we have evaluated the antiviral activities of total aqueous extracts from *Epimedium koreanum* Nakai against a wide array of viruses *in vitro* and *in vivo.* Additionally, we have confirmed the immune-modulatory potential that regulates the innate immune response of *Epimedium koreanum* Nakai. In addition, we tried to identify the active molecules present in the aqueous fraction. Finally, the prophylactic efficacies of *Epimedium koreanum* Nakai against divergent influenza A subtypes, including {A/Aquaticbird/Korea/W81/2005(H5N2)},{A/PR/8/34(H1N1)},{A/Aquaticbird/Korea/W44/2005(H7N3)} and {A/Chicken/Korea/116/2004(H9N2)} were evaluated in a BALB/c murine infection model.

## 2. Materials and Methods

### 2.1. Plant Materials and extract Preparation

A water-soluble herbal extract of *Epimedium koreanum* Nakai was prepared by the Herbal Medicine Improvement Research Center, Korea Institute of Oriental Medicine, Daejeon, Republic of Korea. Medicinal herb, the dried bark of the plant, was obtained from Yeongcheon Oriental Herbal Market (Yeongcheon, Korea) and verified by Professor Ki Hwan Bae at the College of Pharmacy, Chungnam National University. In detail, 100 g of the dried bark was placed in 1 L of distilled water and extracted by heating for 2.5 h at 105 °C using a medical heating plate (Gyeongseo Extractor Cosmos-600, Incheon, Korea). After the extraction, *Epimedium koreanum* Nakai was filtered using a filter paper (0.45 μm) (Millex^®^, Darmstadt, Germany) and stored at 4 °C for 24 h. The extract was then centrifuged at 12,000 rpm for 15 min. The supernatant was collected, and the pH was adjusted to 7.0. The total aqueous extract was then subjected to membrane syringe filtration (0.22 μm) (Millex^®^, Darmstadt, Germany) and lyophilized. The final yield of the water extract of *Epimedium koreanum* Nakai was adjusted to 0.1 mg/mL with phosphate buffered saline (PBS) and stored at 4 °C until administration.

### 2.2. Cells and Viruses

RAW264.7 (ATCC TIB-71), HEK293T (ATCC-11268), MDCK (ATCC CCL-34, NBL-2) and Vero (ATCC CCL-81) cells were grown in DulbeccoCC CCL-81) cells were grow (DMEM, Invitrogen, Carlsbad, CA, USA) supplemented with 10% fetal bovine serum (FBS) (Gibco, Grand Island, NY, USA) and 1% antibiotic/antimycotic solution (Gibco, Grand Island, NY, USA) at 37 ° Grand Isla_2_ concentration of 5%. Green Fluorescent Protein (GFP)-tagged Influenza A (A/PuertoRico/8/34(H1N1)(PR8-GFP), Newcastle Disease Virus (NDV-GFP) and challenge Influenza viruses [{A/Aquaticbird/Korea/W81/2005(H5N2)}, {A/PR/8/34(H1N1)}, {A/Aquaticbird/Korea/W44 /2005(H7N3)}, and {A/Chicken/Korea/116/2004(H9N2)}] were propagated in the allantoic fluid of 10-day-old chicken embryos, and Vesicular Stomatitis Virus (VSV-GFP) and Herpes Simplex Virus (HSV) were propagated on confluent Vero cells. The authors received the Green Fluorescence Protein (GFP)-tagged PR8, NDV, VSV and HSV viruses from Dr. Jae U. Jung, Department of Molecular Microbiology and Immunology, University of Southern California, USA.

### 2.3. Determination of Effective Concentration (EC_50_) of Epimedium koreanum Nakai in Vitro

RAW264.7 and HEK293T cells were grown in 96-well plates (2.5 × 10^4^ cells/well and 2 × 10^4^ cells/well, respectively) and incubated at 37 °C in a 5% CO_2_ atmosphere. After 12 hours, the medium was replaced with two-fold serially diluted *Epimedium koreanum* Nakai (50 aL/well). At 12 hour post treatment (hpt), the cells were washed with PBS once and infected using DMEM containing 1% FBS. RAW264.7 cells were infected with PR8-GFP (MOI = 1.0), VSV-GFP (MOI = 1), NDV-GFP (MOI = 3.0) or HSV-HFP (MOI = 3.0), and HEK293T cells were infected with VSV-GFP (MOI = 0.005) or HSV-GFP (MOI = 2.0) viruses. At 2 hour post infection (hpi), the inocula were removed, washed with PBS once and replaced with DMEM containing 10% FBS. The experiments were performed in triplicate. GFP expression was measured 24 hpi with the Glomax multi-detection system (Promega, WI, USA), according to the manufacturer’s instructions. Graphs were developed for the different cell lines infected with individual viruses based on the dilutions and the GFP expression values. The EC_50_ values were then calculated as the extract concentration yielding 50% GFP expression.

### 2.4. Determination of the Cytotoxic Concentration (CC_50_) of Epimedium koreanum Nakai in Vitro

The CC_50_ was evaluated in a cell viability assay through the trypan blue exclusion test as described elsewhere [21]. The assay was performed using 72-well tissue culture plates. Increasing concentrations (1–160 µL/mL or 0.1–16 µg/mL) of the plant extract were added to confluent RAW264.7 and HEK293T cell monolayers. After 24 h, the cell viability was determined by trypan blue exclusion test. Clarified cells from each treatment group were mixed with 0.4% trypan blue stain (Invitrogen, USA) at a 1:1 ratio. After staining, 10 µL of the mixture was applied to a hemocytometer to obtain the percentages of viable cells; the total number of viable/live cells per mL of aliquot was divided by the total number of cells/mL of aliquot multiplied by 100. Cell counting was done thrice. A graph of the concentrations of the extract as a function of cell viability was developed, and the CC_50_ was calculated as the concentration of the extract resulting in 50% cell viability. The experiment was performed in triplicate.

### 2.5. Antiviral Assays in Epimedium koreanum-Treated RAW264.7 and HEK293T Cells

A viral replication inhibition assay was performed according to Moon *et al.*, [22], with some modifications. RAW264.7 cells were grown in 12-well tissue culture plates (8 × 10^5^ cells/well) and incubated at 37 °C for 12 h. Simultaneously, HEK293T cells were cultured in six-well tissue culture plates (1 × 10^6^ cells/well) under similar conditions. DMEM alone (untreated and virus-only groups), DMEM with 1000 U of recombinant mouse/human interferon (IFN)-β (positive control, Sigma, St. Louis, Missouri, USA) and DMEM with 1.0 μg/mL (10 μL/mL or 1%) of *Epimedium koreanum* Nakai were incubated in different wells for the pre-treatment assay. At 12 h post-treatment (hpt), all of the wells were gently washed with phosphate-buffered saline (PBS) before infection. RAW264.7 cells were infected with either VSV-GFP (MOI = 1.0), PR8-GFP (MOI = 1.0), NDV-GFP (MOI = 3.0) or HSV-GFP (MOI = 3.0), using DMEM supplemented with 1% FBS. Additionally, HEK293T cells were infected with VSV-GFP (MOI = 0.005) and HSV-GFP (MOI = 2.0) viruses. Two hours post-infection (hpi), the unattached viruses were aspirated out with the supernatant, and the wells were gently washed with PBS. Then, DMEM supplemented with 10% FBS and 1% antibiotic/antimycotic solution was added to the wells. GFP expression, which reflects virus replication, was observed at 24 h post infection (hpi) at 200× magnification. The virus titration and cell viability were determined at both 12 and 24 hpi. The cell viability was determined via trypan blue exclusion, and the cell counts were performed in triplicate.

### 2.6. NDV-GFP mRNA Expression and Virus Titration in RAW 264.7 Cells

The total mRNA from RAW264.7 cells was extracted and amplified to estimate the NDV-GFP mRNA expression level [23]. RAW264.7 cells were cultured in 12-well tissue culture plates (8 × 10^5^ cells/well) and incubated for 12 h. The medium was replaced with DMEM alone (untreated and virus-only groups) or DMEM with 1.0 μg/mL (10 μL/mL or 1%) *Epimedium koreanum* Nakai. Twelve hours post-treatment, the cells were infected with NDV-GFP (MOI = 3) and harvested at 0, 6, 12, and 24 hpi. The total mRNA was extracted using the RNeasy Mini Kit (Qiagen, Seoul, Korea) and then converted to cDNA, and PCR was then performed using specific primers. For the APMV-1 M gene, the forward primer was 5′- -TCGAGICTGTACAATCTTGC-3 and the reverse primer was 5′- GTCCGAGCACATCACT GAGC-3′. For the GAPDH, the forward primer was 5′-TGACCACAG TCCATGCCATC-3′ and the reverse primer was 5′-GACGGACACATTGGG GGTAG-3′ [24]. Equal amounts of the PCR products were run on 1.5% ethidium bromide agarose gels and visualized using a GelDoc Imaging System (Bio-Rad, Seoul, Korea). Finally, the relative band intensity (RBI) of the matrix gene compared with that of GAPDH was determined using the GelDoc Imaging System Band Quantification Software (Bio-Rad).

### 2.7. Virus Titration of Treated Cell Supernatants and Infected Cells

The viral titers were measured by plaque assays using Vero cells [25]. Briefly, RAW264.7 and HEK293T cells were cultured in six-well tissue culture plates (1 × 10^6^ cells/well and 8 × 10^5^ cells/well, respectively) and incubated for 12 h. The medium was replaced with DMEM alone (untreated and virus-only groups), DMEM with 1000 U of recombinant mouse/human interferon (IFN)-MEM alone with 1.0 μg/mL (10 μL/mL or 1%) *Epimedium koreanum* Nakai. Twelve hours post-treatment, RAW264.7 cells were infected with PR8-GFP (MOI = 1.0), VSV-GFP (MOI = 1.0), NDV-GFP (MOI = 3.0) or HSV-GFP (MOI = 3.0), and HEK293T cells were infected with VSV-GFP (MOI = 0.005) and HSV-GFP (MOI = 2.0) viruses. For the titration of VSV-GFP, supernatants from each group were collected at 12 and 24 hpi and serially diluted. Independently, VERO cells were cultured in 12-well plates, and when the cell confluency was approximately 75%–80%, the cells were infected with 500 μL of each dilution. Following 2 h incubation at 37°C, the inoculum was removed and replaced with agar (0.45 g/ 20 ml DW). The plates were then incubated for another 46 h at 37 °C and examined for plaque formation at 200х magnification. The viral titers were calculated using the number of plaque-forming units and the dilution factor. In the case of PR8-GFP and HSV-GFP titration, instead of the cell supernatant, infected cells from each group were harvested at 12 and 24 hpi and subjected to five cycles of freezing at −70 °C and thawing at room temperature. The cells were then re-suspended with 500 µL of phosphate-buffered saline (PBS) and serially diluted before being used to infect Vero cells.

### 2.8. Detection of IFN-β and Pro-Inflammatory Cytokines in Epimedium koreanum Nakai-Treated RAW264.7 and HEK293T Cells by Enzyme-Linked Immunosorbent Assay (ELISA)

The pro-inflammatory cytokine inducing effect of *Epimedium koreanum* Nakai *in vitro* was examined using commercial ELISA kits. In the case of RAW264.7 cells, murine interleukin (IL)-6 (BD Bioscience, USA), and IFN-β (PBL Interferon Source, USA) were measured, as previously described [26]. Briefly, RAW264.7 cells were cultured in 6-well tissue culture (TC) plates (1 × 10^6^ cells/well). After 12 h, the cells were treated with 1000 units/mL recombinant murine IFN-β (Sigma-Aldrich) and 1.0 μg/mL (10 μl/mL or 1% v/v) *Epimedium koreanum* Nakai in DMEM containing 10% FBS or medium alone and then incubated at 37 °C with 5% CO_2_. Supernatants were harvested at 0, 12 and 24 hpt, clarified by centrifugation at 2500× g for 10 min at 4 °C and dispensed into murine IFN-β ELISA plates or murine IL-6 capture antibody-coated ELISA plates. In the case of HEK293T cells (cell count: (1 × 10^6^ cells/well), recombinant human IFN-β (Sigma-Aldrich) was used as the positive control and the clarified supernatant was dispensed into commercial human IFN-β (TFB, Inc., Tokyo, Japan) and human IL-6 (Invitrogen, Carlsbad, California, USA) ELISA plates. Murine IFN-β, human IFN-β and human IL-6 ELISA were performed in duplicate, and murine IL-6 ELISA was performed in triplicate.

### 2.9. Determination of the Level of mRNA Induction by Epimedium koreanum Nakai in Vitro by Real-Time PCR Analysis

RAW264.7 and HEK293T cells were grown in six-well tissue culture (TC) plates (1 × 10^6^ cells/well) and incubated at 37 °C; the cells were treated with DMEM + 10% FBS alone (negative control), DMEM with 1000 units/mL recombinant murine/human IFN-β), DMEM with 1.0 μg/mL (10 μl/mL or 1%) *Epimedium koreanum* Nakai, and the cells were harvested at 0, 3, 6, 12, and 24 hpt. The total RNA from the cells was isolated using the RNeasy Mini Kit (Qiagen, Seoul, Korea), and cDNA synthesis was performed using reverse transcriptase (Toyobo, Japan). The different levels of cDNA were quantified by real-time polymerase chain reaction (PCR) using a QuantiTect SYBR Green PCR kit (Qiagen, Seoul, Korea) on a Mygenie96 thermal block (Bioneer, Korea). The PCR primers are listed in Table 2 and Table 3.

### 2.10. Immunoblot Analysis to Determine the Effect of Epimedium koreanum Nakai on Type I IFN-Related Protein Phosphorylation in RAW264.7 Cells

RAW264.7 cells were cultured in six-well tissue culture (TC) plates (1 × 10^6^ cells/well) and incubated at 37 °C. After 12 hours the cells were treated with DMEM + 10% FBS alone (negative control), DMEM with 100 ng/mL LPS (positive control), or DMEM with 1.0 μ0/mL (10 μL/ml or 1%) *Epimedium koreanum* Nakai, and the cells were harvested at 0, 8, 12, and 24 hpt. The cell pellets were washed with phosphate-buffered saline (PBS) and subjected to immunoblot analysis. Briefly, the cell pellets were lysed in radio-immunoprecipitation assay (RIPA) lysis buffer (50 mM Tris-HCl (pH 8.0), 150 mM NaCl, 0.5% sodium deoxycholate, 1% IGEPAL, 1 mM NaF, 1 mM Na3VO4, and 1 ug/mL each of aprotinin and leupeptin). The samples were separated by SDS-PAGE and transferred onto a PVDF membrane (BioRad) in buffer containing 30 mM Tris, 200 mM glycine, and 20% methanol for 2 h. The membranes were blocked for 1 h in Tris-buffered saline containing 0.05% Tween 20 and 5% bovine serum albumin and were then probed with the target protein antibody in 5% FBS-TBST. These incubations were performed at 4 °C overnight with anti-IRF3 (Abcam, #ab25950), anti-phopho-IRF3 (Ser 396), (Cell Signaling, #4947), anti-p65 (Cell Signaling, #4764S), anti-phopho-p65 (Cell Signaling, #3031S), anti-STAT1 (Cell Signaling, #9175), anti-phospho-STAT1 (Cell Signaling, #9167), anti-TBK1 (Cell Signaling, #3504S), or anti-phospho-TBK1 (Cell Signaling, #5483S), anti-p38 (Cell Signaling, #9212), anti-phopho-p38 (Cell Signaling #4631S), anti-ERK (Cell Signaling, #9102), anti-phospho-ERK (Cell Signaling, #9102S), or anti-B-actin (Santa Cruz SC 47778) antibodies. After three 10-min washes with Tris-buffered saline containing 0.05% Tween 20, the membranes were incubated with a horseradish peroxidase-conjugated secondary antibody for 1 hour at room temperature. After three 10-min washes with PBST, the HRP reaction was visualized with the enhanced chemiluminescence detection system (ECL-GE Healthcare) using a Las-3000 mini Lumino Image Analyzer.

### 2.11. Oral Inoculation of Epimedium koreanum Nakai and Viral Challenge in BALB/c Mice

Fifty-two female, five-week-old BALB/c mice were divided into four experimental sets, with two groups per set. Of the four sets, one had two groups with 11 mice each (six for lung virus titration at 3 and 5 days post-infection (dpi)). The remaining three sets had two groups containing five mice each. The mice were orally administered 0.1 mg/mL *Epimedium koreanum* Nakai at a total volume of 200 μL (20 μg per head) 1, 3 and 5 days before infection. The mice in the control groups were orally administered 200 μL of PBS.

The mice were intra-nasally infected with five times the 50% mouse lethal dose (MLD_50_) of H1N1, H5N2, H7N3 or H9N2 in 20 μl of PBS per mouse. Treatment and challenge experiments were conducted in an approved BSL-2 + facility. The body weight and survival were recorded up to 13 dpi. Mice showing a more than 25% body weight loss were considered to have reached the experimental end point and were humanely killed. At 3 and 5 dpi, three mice from each of the two groups from the H1N1-challenged set were randomly sacrificed to measure the lung virus titers.

The animal study was conducted under appropriate conditions with the approval of the Institutional Animal Care and Use Committee of the Bioleaders Corporation, Daejeon, South Korea, Protocol number: BSL-ABLS-13-004. They are in accordance with Institutional, National and International laws for Laboratory Animal Experimentation.

### 2.12. Determination of Lung Viral Titer

Lung tissues from euthanized mice were collected aseptically, and virus titers were determined by 50% tissue culture infectious dose (TCID_50_), as described previously [27]. Briefly, lung tissues were homogenized in 500 mL of PBS containing antibiotics (penicillin, and streptomycin) and antimycotics (Fungizone) compounds (Gibco, Grand Island, NY, USA). Mechanically homogenized lung samples were centrifuged (15 min, 12,000× g and 4 °C) to remove the cellular debris before their storage at −80 °C. Madin-Darby Canine Kidney (MDCK) cells (75%–80% confluent) grown in 96-well microtiter plates were infected with 10-fold serial dilutions (in DMEM containing 1% FBS) of lung homogenate (50 μL/well) in quadruplicate and incubated at 37 °C in a humid atmosphere of 5% CO_2_ for an hour. After absorption, the media was removed, and overlay medium containing l-1-tosylamido-2-phenylethyl chloromethyl ketone (TPCK) trypsin (Thermo Fisher Scientific, Rockford, USA) was added to the infected cells and incubated for 72 h. Viral cytopathic effects (CPE) were observed daily, and the titers were determined by the hemagglutination assay (HA) test indicated as follows. Fifty microliter (50 μL) of 0.5% chicken red blood cells (RBC) was added to 50 μL of cell culture supernatant and incubated at room temperature for 30 min. Wells containing HA were scored as positive. The virus titer was calculated by the Reed and Muench method [28] and expressed as Log_10_ TCID_50_/mL of lung tissues.

### 2.13. Anti-Influenza Effect and Induction of Cytokines by Quercetin on RAW264.7 Cells

Quercetin was kindly provided by Dr. Jin Yeul Ma, Herbal Medicine Improvement Research Center, Korea Institute of Oriental Medicine, Daejeon, Republic of Korea. The main component profile of the water extract of *Epimedium koreanum* Nakai had been analyzed using high-performance liquid chromatography (HPLC) and quercetin had been successfully purified (14). Then, we tested the anti-influenza (PR8-GFP) effect of quercetin upon pre-treatment of the compound. Quercetin was treated at a concentration of 3.0 μg/mL, 12 h before infection with PR8-GFP (MOI = 1.0) and GFP expression was observed at 24 h post infection (hpi) at 200× magnification. Mouse interferon (IFN)-β was treated as the positive control (1000 units/mL). Further, virus titration, secretion of cytokines (IL-6, TNF-α and IFN-β) by quercetin (3.0 μg/mL) in RAW264.7 cells were determined by the methods described in 3.7 and 3.8.

### 2.14. Statistical Analysis

Data are presented as the means ± standard deviations and are representative of at least three independent experiments. Differences between groups were analyzed by analysis of variance (ANOVA), and means were compared by Student’s *t*-test. *p*-values less than 0.05 were regarded as significant. Results for percent initial body weight were also compared by using Student’s *t* test. Comparison of survival was done by log-rank test using GraphPad Prism 6 version.

### 2.15. Biodiversity Rights

Authors have not violated any biodiversity rights during the handling and preparation of *Epimedium koreanum* Nakai and throughout the entire experimental period.

## 3. Results and Discussion

### 3.1. Determination of the Effective Concentration (EC_50_) and Cytotoxic Concentration (CC_50_) of Epimedium koreanum Nakai in Vitro

The EC_50_ can be defined as the extract concentration at which 50% reduction in virus titre is observed, whereas the extract concentration that results in 50% cell viability is considered the CC_50_. To determine the EC_50_ values of *Epimedium koreanum* Nakai against divergent viruses *in vitro*, we developed a modified GFP assay using RAW264.7 and HEK293T cell lines [29,30]. For the reason that, we used only the GFP-tagged viruses, 50% reduction in GFP expression was considered equivalent to the 50% reduction in virus titre.

As shown in Table 1, *Epimedium koreanum* Nakai can inhibit the replication of PR8-GFP (MOI = 1.0), VSV-GFP (MOI = 1.0), NDV-GFP (MOI = 3.0) and HSV-GFP (MOI = 3.0) by 50% at EC_50_ values of 0.94 ± 0.23 µg/mL, 0.82 ± 0.28 µg/mL, 0.49 ± 0.25 µg/mL and 0.62 ± 0.14 µg/mL, respectively. Moreover, extracts inhibited the replication of VSV-GFP (MOI = 0.005) and HSV-GFP (MOI = 2.0) by 50% at EC_50_ values of 1.12 ± 0.31 µg/mL and 1.41 ± 0.41 µg/mL in HEK293T cells (Table 1). Considering these EC_50_ values, we selected 1.0 µg/mL as the optimum dosage of the extract for further *in vitro* antiviral assays based on its effectiveness and convenience during the experiments.

**Table 1:** Determination of EC_50_ and CC_50_ of *Epimedium koreanum* Nakai in RAW264.7 and HEK293T cells.

Cell line	EC_50_ ± S.D.^a ^(µg/mL)				CC_50 _± S.D.^b^ (µg/mL)
	PR8-GFP	VSV-GFP	NDV-GFP	HSV-GFP	
**Raw264.7**	**0.94±0.23**	**0.82±0.28**	**0.49±0.25**	**0.62±0.14**	**14.6±1.68**
**HEK293T**	**-**	**1.12±0.31**	**-**	**1.41±0.41**	**8.4±1.96**

^a^ Effective concentration for 50% reduction in GFP expression. ^b^ Cytotoxic concentration causing 50% cell death. The results are a mean of three independent experiments.

The cytotoxicity of *Epimedium koreanum* Nakai was assessed based on a cell viability test following treatment with various concentrations. *Epimedium koreanum* Nakai had CC_50_ values of 14.6 ± 1.68 µg/mL and 8.4 ± 1.96 µg/mL in RAW264.7 and HEK293T cells, respectively (Table 1). The selection indexes of *Epimedium koreanum* Nakai (SI) for PR8, VSV, NDV and HSV on RAW264.7 cells were 15.5, 17.8, 29.8 and 23.5, respectively and for VSV and HSV on HEK293T cells were 7.5 and 5.9, respectively, suggesting that the extract could be broadly useful as a prophylactic or therapeutic agent.

### 3.2. Inhibitory Effects of Epimedium koreanum Nakai on Viruses in RAW264.7 Cells

**Figure 1:**
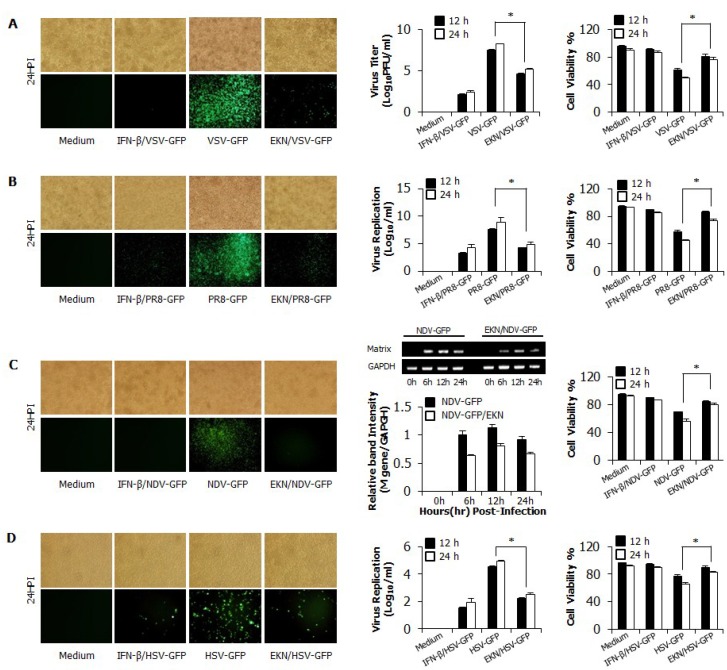
Antiviral activities of *Epimedium koreanum* Nakai in RAW264.7 cells. RAW264.7 cells treated with media alone, 1.0 µg/mL *Epimedium koreanum* Nakai (EKN), or 1000 unit/mL recombinant mouse IFN-β, 12 h prior to infection with (**A**) VSV-GFP; or (**B**) PR8-GFP; or (**C**) NDV-GFP; or (**D**) HSV-GFP at an MOI of 1.0. Images were obtained 24 hpi (200× magnification). Cell viabilities were determined by trypan blue exclusion and presented as a percentage of the control (cells without treatment). Viruses were titrated from the supernatant for VSV-GFP and from the infected cells for PR8-GFP and HSV-GFP, respectively. In the case of NDV-GFP, expression of NDV M-mRNA over time in each treatment group was confirmed by specific PCR primers, which are shown in Table 2. All samples were normalized using GAPDH. Equal amounts of PCR products were run on 1.5% ethidium bromide agarose gels and visualized using the GelDoc Imaging System (**bottom** panel). The relative band intensity (RBI) of M-mRNA expression from the same experiment is shown (**top** panel). RBI was determined (gene/GAPDH) using the GelDoc Imaging System Band Quantification Software. Error bars indicate the range of values obtained from two independent experiments. Cell viabilities are expressed as mean ± SD. Error bars indicate the range of values obtained from counting in triplicate in three independent experiments. (* *p* < 0.05 indicates a significant difference between groups compared by Student’s *t*-test).

To evaluate the *in vitro* antiviral activity of *Epimedium koreanum* Nakai, we checked viral replication with divergent GFP-expressing viruses, including RNA and DNA viruses, in RAW264.7 cells. Viral replication was monitored with GFP-expressing level upon treatment with cytotoxic-free (data not shown) extracts. A total aqueous extract of *Epimedium koreanum* Nakai-treated RAW264.7 cells (1 μg/mL (1% v/v)) exhibited a marked reduction in GFP expression, whereas the untreated groups had high levels of GFP expression for VSV (Figure 1A), PR8 (Figure 1B), NDV (Figure 1C) and HSV (Figure 1D). When quantitated, extract-treated cells showed a significant reduction in GFP expression compared to the untreated group (data not shown). These results correlate with the viral titers of VSV-GFP in the cell supernatant and the viral titers of PR8-GFP and HSV-GFP in infected cells. *Epimedium koreanum* Nakai treatment reduced the viral titers by nearly 1.5-fold, 1.8-fold and 2-fold against VSV-GFP, PR8-GFP and HSV-GFP at 24 hpi, respectively (Figure 1A–C). Importantly, *Epimedium koreanum* Nakai-treated cells displayed a significant reduction in cell death following infection with all tested viruses compared with the untreated cells (Figure 1A–D). In the case of NDV, we measured the mRNA expression of the NDV M gene via RT-PCR to estimate the replication (Figure 1C right panels) of NDV-GFP. As expected, the expression of the Matrix gene mRNA in the extract-treated cells was decreased in a time-dependent manner compared with the untreated group at 6–24 hpi. These results clearly show evidence that the total aqueous extract of *Epimedium koreanum* Nakai is able to reduce the replication of the VSV, PR8, NDV and HSV viruses in RAW264.7 cells.

### 3.3. Inhibitory Effects of Epimedium koreanum Nakai on Viruses in HEK293T Cells

**Figure 2:**
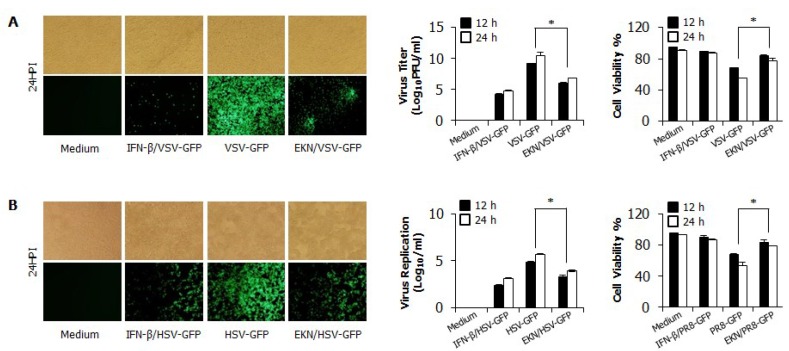
Antiviral activities of *Epimedium koreanum* Nakai in HEK293T cells. HEK293T cells treated with media alone, 1.0 µg/mL *Epimedium koreanum* Nakai (EKN), or 1000 unit/mL recombinant human IFN-β, 12 h prior to infection with (**A**) VSV-GFP; or (**B**) HSV-GFP at an MOI of 1.0 and 3.0, respectively. Images were obtained at 24 hpi (200 × magnification). Viruses were titrated from the cultured supernatant and from the infected cells for VSV-GFP and HSV-GFP, respectively. Virus titrations are expressed as mean ± SD. Error bars indicate the range of values obtained from two independent experiments. Cell viabilities were determined by trypan blue exclusion and presented as a percentage of the control (cells without treatment). Cell viabilities are expressed as mean ± SD. Error bars indicate the range of values obtained from counting in triplicate in three independent experiments (* *p* < 0.05 indicates a significant difference between groups compared by Student’s *t*-test).

We then determined the antiviral activity of *Epimedium koreanum* Nakai in Human Embryonic Kidney (HEK293T) cells. Similarly; anti-viral activity was observed with GFP-tagged VSV and HSV viruses upon pre-treatment with the extract. As shown in Figure 2A,B; extract-treated HEK293T cells exhibited markedly reduced GFP expression and virus titers compared with the untreated groups; which presented high levels of GFP expression and virus replication. Moreover, extract-treated HEK293T cells showed a significant reduction in cell death following infection with all tested viruses compared with the untreated cells. These results clearly indicate that the total aqueous extract of *Epimedium koreanum* Nakai also reduce the replication of RNA or DNA viruses in epithelial cells.

### 3.4. Detection of IFN-β and Pro-Inflammatory Cytokines by Epimedium koreanum Nakai in Vitro

**Figure 3:**
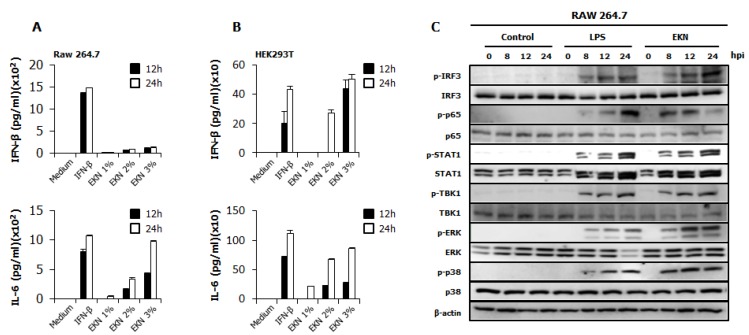
Induction of cytokines and the phosphorylation of the signal molecules by *Epimedium koreanum* Nakai *in vitro*. (**A**) RAW264.7; and (**B**) HEK293T cells were treated with DMEM containing 10% FBS alone, with 1000 unit/mL recombinant mouse or human IFN-β, or with 1.0 μg/mL *Epimedium koreanum* Nakai (EKN) and incubated at 37 °C with 5% CO_2_. Supernatant from each group was harvested at 0, 12 and 24 hpt and clarified by centrifugation at 2500× g for 10 min at 4 °C. Clarified supernatants were dispensed into the murine IFN-β and IL-6, and human IL-6 and IFN-β capture antibody-coated ELISA plate to measure cytokine secretion. The test was performed in duplicate for IFN-β, human IL-6 and in triplicate for other cytokines. The data shows representative means ± SD of each murine cytokine measured over time. (**C**) For the determination of Type I IFN-related or NF-κB related protein phosphorylation, cells were harvested at 0, 8, 12, and 24 hpt with LPS or *Epimedium koreanum* Nakai (EKN) and washed with phosphate-buffered saline (PBS) and subjected to immunoblot analysis. The samples were separated by SDS-PAGE, transferred onto a PVDF membranes and were probed with the target protein antibodies (anti-IRF3/anti-phopho-IRF3, anti-p65/anti-phopho-p65, anti-STAT1/anti-phopho-STAT1, anti-TBK1/anti-phopho-TBK1, anti-p38/anti-phopho-p38, anti-ERK/anti-phopho-ERK, anti-β-actin) before visualizing with the enhanced chemiluminescence detection system (ECL-GE healthcare) using a Las-3000 mini lumino-image analyzer.

To elucidate the possible mechanism of the antiviral activities of *Epimedium koreanum* Nakai, we measured the levels of interferon-β (IFN-β) and the pro-inflammatory cytokine that is secreted from the extract-treated supernatant, on RAW264.7 and HEK293T cells (Figure 3). As shown in Figure 3A, *Epimedium koreanum* Nakai induced high levels of secreted IL-6 at both 12 hpt and 24 hpt compared with IFN-β-treated cells in a concentration-dependent manner and also secreted significantly higher levels of IFN-β, although the secreted level was not as high as the levels obtained from the IFN-β-treated cells. Moreover, *Epimedium koreanum* Nakai induced the secretion of IFN-β and IL-6 in HEK293T cells (Figure 3B) and the secreted amount was significantly high, and in line with human IFN-β-treated positive controls. These results suggest that *Epimedium koreanum* Nakai can stimulate immune cells and epithelial cells and induce the secretion of IFNs and pro-inflammatory cytokines that may mediate the antiviral state in cells.

### 3.5. Epimedium koreanum Nakai Induces the Activation of Signal Molecules in the Type I IFN Signaling Pathway

The antiviral response of *Epimedium koreanum* Nakai may relate to the innate immune response through the expression of cytokines, such as IL-6 and IFN-β. To correlate these observations with the IFN-inducing signaling pathway, we examined the phosphorylation of interferon related signal molecules and p65 phosphorylation related to NF-kB activation. For this, immunoblot analyses were performed using whole cell lysates of extract-treated RAW264.7 cells. As shown in Figure 3C, *Epimedium koreanum* Nakai significantly upregulated the phosphorylation of IRF-3, STAT1, TBK1, p65, p38 and ERK, which are key signaling molecules belonging to the type I interferons (IFNs) and NF-κB pathways. The phosphorylation of IRF3 is a key indicator of interferon signal transduction. Upon virus infection, the phosphorylated IRF3 translocated into the nucleus and initiated the transcription of type I interferons (IFNs). Consequently, the produced type I interferons (IFNs) binds to the JAK-STAT pathway, leading to the phosphorylation of STAT1 and the transcriptional activation of Interferon-stimulated gene (ISGs). These activated ISGs are then involved in controlling viral infection. Our results clearly demonstrate that treatment with the *Epimedium koreanum* Nakai extract can induce potent IRF3 phosphorylation at 8 hpt, the effect of which markedly increases with time. Furthermore, this increased STAT1 phosphorylation indicates the active functions of the ISGs. In addition to the activation of type I interferons (IFNs), the extract-treated RAW264.7 cells were able to elicit obvious activation of NF-κB (P65), leading to strong secretion of pro-inflammatory cytokines. The phosphorylation of these molecules induced by extracts is comparable to that obtained with LPS treatment, which is a known potent stimulator of TLR4.

### 3.6. Epimedium koreanum Nakai Induces Antiviral Gene Expression in the Type I IFN Signaling Pathway

**Figure 4:**
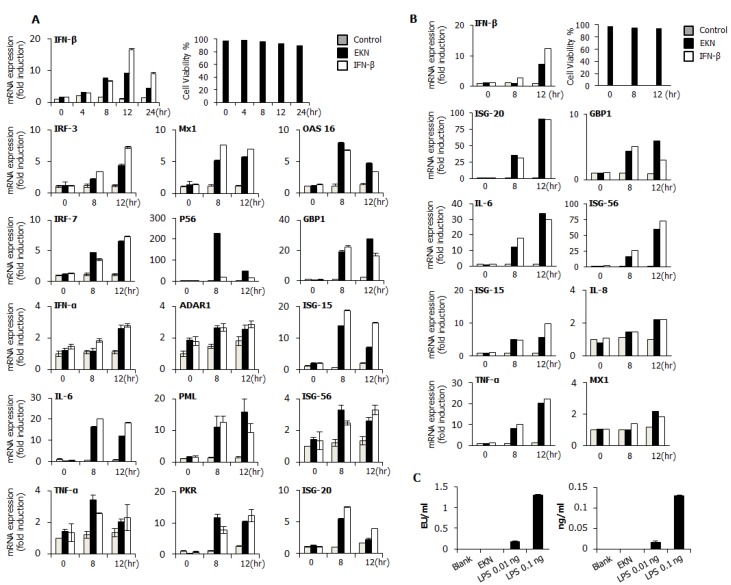
Induction of IFN-β, IFN-related gene and ISG’s transcripts by *Epimedium koreanum* Nakai *in vitro*. RAW264.7 and HEK293T cells were treated with DMEM + 10% FBS alone, *Epimedium koreanum* Nakai (EKN) (1.0 μg/mL), or 1000 units/mL of recombinant murine or human IFN-β. The time-dependent changes in mRNA expression after treatment in (**A**) RAW264.7; and (**B**) HEK293T cells were confirmed by real-time PCR using the primers shown in Table 2 and Table 3. Real-time PCR was carried out with the use of a QuantiTect SYBR Green PCR kit (Qiagen) on a Mygenie96 thermal block (Bioneer). Error bars indicate the range of values obtained from two independent experiments. (**C**) *Epimedium koreanum* Nakai was tested for residual endotoxin contamination using a limulus amebocyte lysate (LAL) assay and was found to not be contaminated with endotoxin. (Cell viabilities were determined by trypan blue exclusion and are expressed as mean ± SD).

We further evaluated the induction of different antiviral and interferon-stimulatory genes at the transcription level in response to *Epimedium koreanum* Nakai treatment in RAW264.7 and HEK293T cells. Cells were treated with *Epimedium koreanum* Nakai at a concentration of 1.0 μg/mL (10 μL/mL or 1%). As confirmed by real-time PCR, the mRNA expression levels of various antiviral and interferon stimulatory genes were up-regulated to levels similar to those found with the IFN-β-treated positive controls (Figure 4). Initially, to determine the transcription levels of various antiviral genes in *Epimedium koreanum* Nakai-treated RAW264.7 cells from 0 hpt to 24 hpt, an IFN-β real-time PCR assay was performed to monitor the time-dependent mRNA changes. After normalization to GAPDH, the extract-treated cells displayed a seven-fold increase in the level of IFN-β mRNA at 8 hpt and a nine-fold induction level at 12 hpt compared with untreated cells, respectively (Figure 4A).

Therefore, we performed a PCR assay for other genes of interest at 0, 8 and 12 hpt using specific primers (Table 2 and Table 3) in both RAW264.7 and HEK293T cells. We found that the transcriptional levels of various antiviral genes were up-regulated by *Epimedium koreanum* Nakai at 12 hpt, including Mx1, GBP-1 and PML to levels that were 5.6-fold, 28-fold and 15-fold higher, respectively, than those of the control in RAW264.7 cells. Moreover, at 8hpt, the IL-6 and OAS-16 transcriptional levels were observed to be up-regulated by 15-fold and 8-fold, respectively. In addition to the elevated levels of IFN-β, the extract induced the transcription of interferon-stimulatory genes (ISGs), such as ISG-15 and ISG-56 (14-fold and 5-fold), respectively at 8 hpt in RAW264.7 cells. The observed elevated transcriptional patterns for some of the antiviral genes were similar to the pattern of the IFN-β-treated positive controls. Furthermore, similar transcriptional activation patterns were observed in extract treated HEK293T cells. Interestingly, the highest fold inductions of cellular transcriptional levels were observed at 12 hpt for all the primers. Transcriptional levels of extract-treated HEK293T cells for IFN-β, GBP-1, IL-6, IL-8, ISG-15, Mx-1 and TNF-α were up-regulated by 8-fold, 5-fold, 30-fold, 2-fold, 5-fold and 2-fold, respectively. Importantly, highest transcriptional induction levels of 90-fold and 60-fold were observed for ISG-20 and ISG-56, respectively. All these transcriptional patterns were similar to the pattern of human IFN-β-treated positive control. The overall results suggest that *Epimedium koreanum* Nakai has the capacity to up-regulate the transcription levels of IFN-β, interferon-stimulating genes (ISGs) and various antiviral genes. This molecular-level activation may have a direct relationship with the extract’s antiviral abilities, which were observed in both RAW264.7 and HEK293T cells.

**Table 2:** Mouse primer sets used to confirm mRNA expression.

Gene	Primers	
	Forward	Reverse
IFN-β	5’-TCCAAGAAAGGACGAACATTCG-3’	5’-TGCGGACATCTCCCACGTCAA-3’
Mx1	5’-ACAAGCACAGGAAACCGTATCAG-3’	5’-AGGCAGTTTGGACCATCTTAGTG-3’
IRF-3	5'-GTGCCTCTCCTGACACCAAT-3'	5'-CCAAGATCAGGCCATCAAAT-3'
IRF-7	5'-AAGCTGGAGCCATGGGTATG-3'	5'-GACCCAGGTCCATGAGGAAG-3'
P-56	5'-CCCACGCTATACCATCTACC-3'	5'-CTGAGGCTGCTGCTATCC-3'
GBP-1	5'-AAAAACTTCGGGGACAGCTT-3'	5'-CTGAGTCACCTCATAAGCCAAA-3'
PML	5'-CCTGCGCTGACTGACATCTACT-3'	5'-TGCAACACAGAGGCTGGC-3'
ADAR-1	5'-CCAAAGACACTTCCTCTC-3'	5'-CAGTGTGGTGGTTGTACT-3'
PKR	5'-GCCAGATGCACGGAGTAGCC-3'	5'-GAAAACTTGGCCAAATCCACC-3'
OAS-16	5'-GAGGCGGTTGGCTGAAGAGG-3'	5'-GAGGAAGGCTGGCTGTGATTGG-3'
ISG-15	5’-CAATGGCCTGGGACCTAAA-3’	5’-CTTCTTCAGTTCTGACACCGTCAT-3’
ISG-20	5'-AGAGATCACGGACTACAGAA-3'	5'-TCTGTGGACGTGTCATAGAT-3'
ISG-56	5’-AGAGAACAGCTACCACCTTT-3’	5’-TGGACCTGCTCTGAGATTCT-3’
IFN-a	5'-ATAACCTCAGGAACAACAG-3'	5'-TCATTGCAGAATGAGTCTAGGAG-3'
TNF-α	5’-AGCAAACCACCAAGTGGAGGA-3’	5’-GCTGGCACCACTAGTTGGTTGT-3’
IL-6	5'-TCCATCCAGTTGCCTTCTTGG-3'	5'-CCACGATTTCCCAGAGAACATG-3'
GAPDH	5’-TGACCACAGTCCATGCCATC-3’	5’-GACGGACACATTGGGGGTAG-3’

**Table 3:** Human primer sets used to confirm mRNA expression.

Gene	Primers	
	Forward	Reverse
**IFN-β**	5’-CATCAACTATAAGCAGCTCCA-3’	5’-TTCAAGTGGAGAGCAGTTGAG-3’
**MX-1**	5'-CCAAAGACACTTCCTCTC-3'	5'-CAGTGTGGTGGTTGTACT-3'
**GBP-1**	5'-AGAGATCACGGACTACAGAA-3'	5'-TCTGTGGACGTGTCATAGAT-3'
**ISG-15**	5'- GAG AGG CAG CGA ACT CAT CT -3'	5'- CTT CAG CTC TGA CAC CGA CA -3'
**ISG-20**	5′-CTCCTGCACAAGAGCATCCA-3′	5′-CGTTGCCCTCGCATCTTC-3′
**ISG-56**	5′-AAGGCAGGCTGTCCGCTTA-3′	5′-TCCTGTCCTTCATCCTGAAGCT-3′
**IL-8**	5'-CTCTCTTGGCAGCCTTCCTGATT-3'	5'-AACTTCTCCACAACCCTCTGCAC-3'
**IL-6**	5'-CCACACAGACAGCCACTCACC-3'	5'-CTACATTTGCCGAAGAGCCCTC-3'
**TNF-α**	5' -ATGAGCACTGAAAGCAT-3'	5'-TCGACGGGGAGTCGAACT-3'
**β-actin**	5'-CCAACCGCGAGAAGATGACC-3'	5'-GATCTTCATGAGGTAGTCAGT-3'

### 3.7. Protection Against Diverse Influenza A Virus Infection by Oral Administration of Epimedium koreanum Nakai in Balb/c Mice

**Figure 5:**
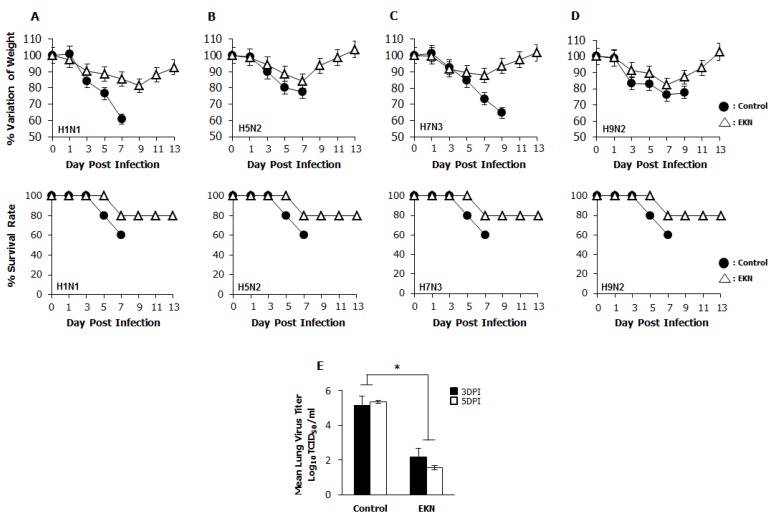
Oral administration of *Epimedium koreanum* Nakai provides protection against lethal infection with divergent influenza A subtype in BALB/c mice. 5-week-old female BALB/c mice were orally administered with 0.1 mg/mL *Epimedium koreanum* Nakai (EKN) in a total volume of 200 µL (20 µg/head) at 1, 3 and 5 days before infection with 5 MLD_50_ (**A**) H1N1; (**B**) H5N2; (**C**) H7N3; and (**D**) H9N2 Influenza A sub types. Control groups were orally administered with 200 µL of PBS. Percentage variation of weight, and percentage survival after challenge were recorded until 13 dpi. (E) Virus titers in lung tissues of the H1N1 infected mice were measured by TCID_50_ at 3 and 5 dpi. (* *p* < 0.05 indicates a significant difference between groups compared by Student’s *t*-test).

To confirm the prophylactic effects of *Epimedium koreanum* Nakai against diverse influenza A viral infection, after oral inoculation of extracts, groups of BALB/c mice were infected with 5 times of 50% mouse lethal dose (MLD_50_) of the A/PR/8/34(H1N1), A/Aquaticbird/Korea/W81/2005(H5N2), A/Aquatic bird/Korea/W44/2005(H7N3) or A/Chicken/Korea/116/2004(H9N2) influenza A subtypes. The mice were orally treated with *Epimedium koreanum* Nakai at 20 µg per head in a total volume of 200 µL before infection with lethal doses of influenza A subtypes. A minimum effective dose of 20 µg per head was chosen based on our previous *in vivo* experimental experiences with various herbal extracts (data not shown). After the challenge, the untreated (PBS) groups were observed to have severe illnesses and the body weights were found to decrease progressively. Moreover, the control group succumbed to death by 9 days post infection (dpi), regardless of the virus used for the infection. In contrast, the *Epimedium koreanum* Nakai-treated mice showed a ≤20% body weight loss between 5 and 7 dpi and had begun to recover their lost weight by 8 dpi, returning to their normal state by 13 dpi (Figure 5). Furthermore, all the groups that were orally inoculated with the herbal extract pre-infection had similar protection levels: 80% for all of the influenza A subtypes tested (Figure 5).

The ability of *Epimedium koreanum* Nakai to inhibit viral replication in the lung tissues of infected mice was evaluated in an H1N1-infected experimental set. Three mice from each group were sacrificed, and their lungs were collected 3 and 5 days post-infection for viral titration. Overall, the oral inoculation of *Epimedium koreanum* Nakai reduced the viral titers in the lungs of the infected mice in the extract-treated groups compared with the untreated controls, which had lung viral titers of 5.13 log TCID_50_/mL and 5.35 log TCID_50_/mL on 3 dpi and 5 dpi, respectively. Interestingly, the extract-treated groups had significantly reduced viral titers 2.1 log TCID_50_/mL and 1.5 log TCID_50_/mL, at 3 dpi and 5 dpi, respectively (Figure 5E).

Taken together, these results indicate that *Epimedium koreanum* Nakai induced the antiviral state, which is sufficiently strong to inhibit viral replication and promoted the survival of mice against lethal infections of diverse influenza A viruses.

### 3.8. Inhibitory Effect of Quercetin on Influenza Virus (PR8-GFP) and Induction of IFN-β or Pro-Inflammatory Cytokines in RAW264.7 Cells

**Figure 6:**
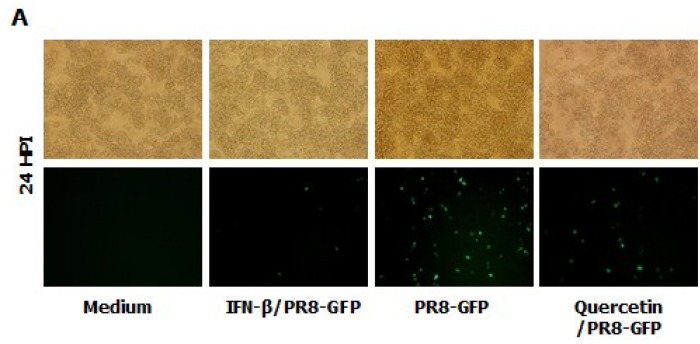

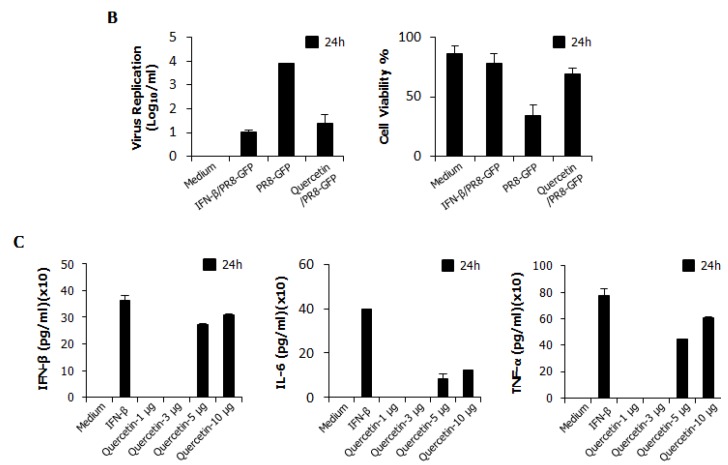
Anti-influenza (PR8-GFP) effect of Quercetin and induction of IFN-β and pro-inflammatory cytokines in RAW264.7 cells. (**A**) RAW264.7 cells treated with media alone, 5.0 µg/mL Quercetin, or 1000 unit/mL recombinant mouse IFN-β, 12 h prior to infection with PR8-GFP at an MOI of 1.0 The GFP expression images were obtained at 24 hpi (200× magnification); (**B**). Viruses were titrated from the infected cells for PR8-GFP. Cell viabilities were determined by trypan blue exclusion and are expressed as mean ± SD. (**C**). Supernatant from RAW264.7 cells treated with media alone, with varying concentrations of Quercetin or with 1000 unit/mL recombinant mouse IFN-β were harvested at 24 hpt and tested for secreted murine IFN-β, IL-6 and TNF-α using capture antibody-coated ELISA plates. The test was performed in duplicate for IFN-β and triplicate for other cytokines. The data show the representative means ± SD of each murine cytokine measured over time.

*Epimedium koreanum* Nakai is a natural product and contains many effective components. For a detailed understanding of the main component profile of the water extract of *Epimedium koreanum* Nakai, a high-performance liquid chromatography (HPLC) system was employed. Among marker compounds of *Epimedium koreanum* Nakai, quercetin and icariin have been representatively identified at 270 nm based on comparison to the standard compounds (14). Based on this, we tested the anti-influenza (PR8-GFP) effect of quercetin upon pre-treatment of the compound (5.0 μg/mL). Minimum effective dose of 5.0 μg/mL was chosen based on our preliminary experiments on the efficacy of quercetin (data not shown). Interestingly, treatment with quercetin markedly inhibited virus replication (Figure 6A). The quercetin-treated group displayed reduced GFP expression compared to untreated groups, which had high levels of GFP expression. The observed GFP data correlated with the viral titers where, quercetin treatment reduced the viral titers by nearly 3.5-fold against PR8-GFP at 24 hpi (Figure 6B). Furthermore, treatment of quercetin (5.0 μg/mL) had marked increase in cytokine secretion in RAW264.7 cells (Figure 6B). These data strongly suggest that quercetin, a major constituent of *Epimedium koreanum* Nakai, might be able to induce the antiviral state in cells and subsequent inhibition of virus replication.

### 3.9. Discussion

Because of concerns related with the side effects, higher costs and lack of efficacy of conventional medicines, the use of natural products as alternatives to conventional treatment in the healing and treatment of various diseases has been increasing in the last few decades [31]. Among natural products, medicinal plants serve as viable alternatives, safer choices, or in some cases, as the only effective treatment. Moreover, the use of plants as medicines dates to the earliest years of man’s evolution [31,32,33], and therefore poses less safety-related concerns. A larger number of these plants and their isolated constituents have shown beneficial preventive and therapeutic effects, including anti-oxidant, anti-inflammatory, anti-cancer, anti-bacterial, and immune-modulatory properties [34,35,36,37,38].

Moreover, as substitutes for chemosynthesis drugs and vaccines, medicinal plants show potential against a wide range of viruses, such as vaccinia, vesicular stomatis virus, and Sendai virus *etc.* [39,40,41]. In particular, the anti-influenza virus effects of several herbal extracts have been reported [42,43,44]. Among the promising medicinal plants, *Epimedium koreanum* Nakai is an herb with a rich historical background and can mainly be found wild in mainland China and Korea [45].

In this study, we demonstrated that *Epimedium koreanum* Nakai contains broad spectrum antiviral activity *in vitro* and against divergent subtypes of influenza A virus in BALB/c mice. In the cytotoxicity of antiviral reagents, *Epimedium koreanum* Nakai has been used for human consumption for a long time, and side effects after a dose have rarely been reported. Importantly, *Epimedium koreanum* Nakai did not have any significant cytotoxic effect on the tested cell lines. Moreover, the cell cytotoxic concentration (CC_50_) of *Epimedium koreanum* Nakai was several magnitudes higher than the effective concentrations (EC_50_) and the selection indexes (SI) of *Epimedium koreanum* Nakai for various viruses indicate the higher safety margin of the extract for therapeutic and/or prophylactic purposes. First, we found that the total aqueous extract of *Epimedium koreanum* Nakai inhibited the replication of influenza (Figure 1B), VSV (Figure 1A and Figure 2A), NDV (Figure 1C), and HSV (Figure 1D and Figure 2B) viruses in immune cells (RAW264.7) and epithelial cells (HEK293T). Moreover, oral administration of *Epimedium koreanum* Nakai increased the survival rate of mice subjected to lethal challenges with different influenza A virus subtypes, including H1N1, H5N2, H7N3 and H9N2 (Figure 5). Although *Epimedium koreanum* Nakai-inoculated mice initially displayed little weight reduction, the majority of them did not lose more than 25% of their body weight. In contrast, all of the mice in the control groups displayed more than 25% losses within 9 dpi and were humanely killed. Influenza virus causes a rapid reduction in the body weight of infected mice. Therefore, 25% body weight loss is considered the humane end point for sacrificing influenza virus-infected mice [46]. These results suggest that *Epimedium koreanum* Nakai is sufficiently strong to inhibit viral replication and promoted the survival of mice against lethal infections of diverse influenza A viruses.

After viral infection, the host initially recognizes the infection and rapidly evokes the induction of type I interferons and pro-inflammatory cytokines, generating an anti-viral innate immune response [47]. Induction of the antiviral state at an early point of virus infection is critical to control the spread and pathogenesis of viruses [48]. Likewise, we hypothesized that *Epimedium koreanum* Nakai induces an antiviral state via the induction of type I interferons and pro-inflammatory cytokines and we determined the induction of antiviral, IFN-stimulated genes (ISGs) (Figure 4) and secretion of IFN-β and IL-6 (Figure 3) by *Epimedium koreanum* Nakai *in vitro*.

In fact, interferon and pro-inflammatory cytokine production can have both beneficial and harmful effects depending on the amount, timing and duration of cytokine release. For instance, during pathogenic influenza virus infection, robust cytokine production (cytokine storm), excessive inflammatory infiltrates, and virus-induced tissue destruction all contribute to morbidity and mortality [49]. However, an induced antiviral state by tight regulation will be a very important defense mechanism against virus infection [50]. Interestingly, upon challenge with viruses, a notable pattern of cytokine regulation and secretion was observed in *Epimedium koreanum* Nakai-treated cells, correlating with the observations found in the cell viability assay in this study (Figure 1 and Figure 2).

For a detailed understanding of *Epimedium koreanum* Nakai on the activation of antiviral signaling, we examined the effect of *Epimedium koreanum* Nakai on the phosphorylation of IRF-3, p65, TBK1, STAT1, ERK and p38 which are key signaling molecules present in the type I IFN and NF-κB signaling pathways. Upon stimulation of the PRRs (Pattern Recognition Receptors) or unknown receptors of the host cell by foreign materials containing pathogens, downstream signal transduction activities, including activation of adaptor signal molecules or transcriptional factors, can initiate the induction of type I interferon and pro-inflammatory cytokines to up-regulate the antiviral status of the host cell [51,52]. In this study, we found that *Epimedium koreanum* Nakai treatment can induce the phosphorylation of IRF-3, STAT1 and TBK1 in a time-dependent manner, providing evidence of the downstream signal transduction in the type I IFN signaling pathway (Figure 3C). Additionally, the activation of NF-κB (p65, pERK, p38), which leads to a strong secretion of pro-inflammatory cytokines, could also be observed. This phosphorylation can lead to the rapid production of type I IFNs and various inflammatory cytokines that play a pivotal role in stimulating the antiviral state and subsequent clearance of viruses [53].

Actually, endotoxin (LPS) is a known immunomodulator and is often a contaminant in biological preparations. Thus, one of the principal concerns in the field is that the macrophage-stimulating properties of the herbal extracts may be due to contamination from bacterial endotoxin (LPS or lipid A-associated protein) [54,55]. Therefore, *Epimedium koreanum* Nakai was tested for endotoxin contamination using a Limulus Amebocyte Lysate (LAL assay) assay and was found to be contaminated with only trace amounts of endotoxin (Figure 4C).

RAW264.7 cells (mouse macrophages) are versatile immune system cells that play indispensable roles in both the innate and adaptive immune responses [56]. They exhibit various immune responses to pathogenic challenge, such as phagocytosis, cytokine secretion, antigen presentation, and adherence [57]. Because of their wide range of functions, macrophages have been extensively studied for their significant role in the immune system, particularly in antiviral response [58]. Moreover, the immune-stimulating effects of different substances have been well established in murine macrophage cells [23,59,60,61] and previous studies have elucidated the capacity of murine macrophages to achieve the antiviral state upon successful stimulation [22,62,63]. Consequently, we decided to use murine macrophages to evaluate the antiviral effect of the water-soluble herbal extract from *Epimedium koreanum* Nakai against divergent viruses. In contrast, human embryonic kidney (HEK293T) cells are epithelial cells which are less known for their relationship with the immune system. It is known that HEK293T cells have less prominent pattern recognition receptors (PRRs), especially Toll-like receptors (TLRs) [64]. This suggests that for activating the HEK293T cells, an active compound/s must penetrate the cell membrane and activate the receptors present in the cytoplasm. Therefore, it is clear that aqueous extract of *Epimedium koreanum* Nakai contains the components which can stimulate both the cell surface PRRs and cytoplasmic PRRs. Therefore, *Epimedium koreanum* Nakai which has the ability to modulate both RAW264.7 and HEK293T cells in a beneficial manner indicates its broad antiviral potential.

It has been reported that *Epimedium koreanum* Nakai contains various active components, including flavonoids, flavonol glycosides, alkaloids, polysaccharides and microelements [65,66]. Flavonoids of *Epimedium koreanum* Nakai mainly contain Icariin, Qercetin, Icariside II, Epimedin, Epimedosides, Hyperoside, and Chlorogenic acid. In our previous study, we identified the main component of *Epimedium koreanum* Nakai as quercetin and icariin using HPLC [14]. Quercetin, the major active component of *Epimedium koreanum* Nakai, has been shown to inhibit porcine epidemic diarrhea virus [67,68]. Based on this lead, we tested quercetin for its antiviral activity and immune-modulatory properties. Quercetin displayed striking antiviral properties and induced cytokine secretion (Figure 6) confirming its importance to the observed biological properties. Therefore, the observed antiviral effects of *Epimedium koreanum* Nakai must be due to the presence of quercetin and other active compounds in *Epimedium koreanum* Nakai, and the relationship between mechanisms of antiviral effects and active compounds containing quercetin must be studied further.

In the present study, we noticed that treatment with the total aqueous extract of *Epimedium koreanum* Nakai displayed a striking anti-viral effect both *in vitro,* and in *in vivo* animal models. As the underlying mechanism, valuable components of *Epimedium koreanum* Nakai including quercetin could induce the secretion of type I IFN and pro-inflammatory cytokines, stimulating an antiviral state in the host cell and can be a promising prophylactic agent to inhibit viral infections through its activation. However, the optimum dosage of *Epimedium koreanum* Nakai for practical application and the longevity of the effect within the host should be examined to obtain a better protection against viral infections.

## 4. Conclusions

The present study strongly suggests that the total aqueous extract of *Epimedium koreanum* Nakai has anti-viral effects against VSV-GFP, PR8-GFP, NDV-GFP and HSV-GFP *in vitro* and against divergent influenza A subtypes, such as H1N1, H5N2, H7N3 and H9N2, in the *in vivo* mouse model*.* Moreover, our study indicates that extracts of *Epimedium koreanum* Nakai, which contain quercetin and other active components, can induce the secretion of type I IFN and pro-inflammatory cytokines and the subsequent stimulation of the antiviral state in host cells as a possible mechanism. Thus, the use of *Epimedium koreanum* Nakai as an orally antiviral agent has the potential to be an effective herbal remedy for prophylaxis and therapeutic applications in both humans and livestock.
