# Correction: Won-Kyung Cho; et al. Epimedium Koreanum Nakai Displays Broad Spectrum of Antiviral Activity In Vitro and In Vivo by Inducing Cellular Antiviral State. *Viruses* 2015, *7*, 352–377

**DOI:** 10.3390/v10060304

**Published:** 2018-06-04

**Authors:** Won-Kyung Cho, Prasanna Weeratunga, Byeong-Hoon Lee, Jun-Seol Park, Chul-Joong Kim, Jin Yeul Ma, Jong-Soo Lee

**Affiliations:** 1 Korean Medicine (KM) Based Herbal Drug Development Group, Korea Institute of Oriental Medicine, Daejeon 305-764, Korea; wkcho@kiom.re.kr; 2 College of Veterinary Medicine, Chungnam National University, 220 Gung-Dong, Yuseong-Gu, Daejeon 305-764, Korea; prasannapdn05@gmail.com (P.W.); byeonghoon_2@naver.com (B.-H.L.); pjs123a@naver.com (J.-S.P.); cjkim@cnu.ac.kr (C.-J.K.)

The authors wish to make the following change to their paper [1]. The image showing the immunoblot for p. 38 was not placed correctly in the original Figure 3C [1]. Figure 3 should be replaced with:

The authors would like to apologize for any inconvenience caused. The change does not significantly affect the scientific results. The manuscript will be updated and the original will remain available on the article webpage.

## Figures and Tables

**Figure 3:**
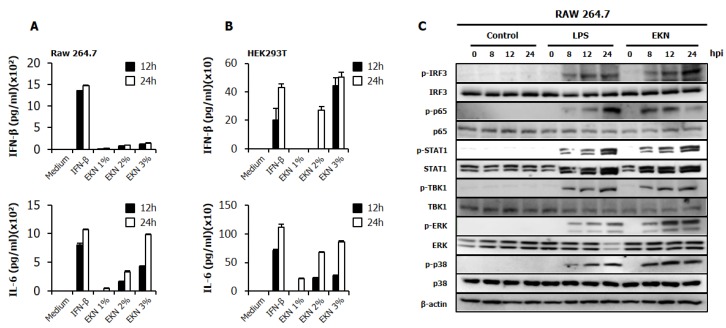
Induction of cytokines and the phosphorylation of the signal molecules by Epimedium koreanum Nakai in vitro. (**A**) RAW264.7; and (**B**) HEK293T cells were treated with DMEM containing 10% FBS alone, with 1000 unit/mL recombinant mouse or human IFN-β, or with 1.0 μg/mL Epimedium koreanum Nakai (EKN). This was incubated at 37 °C with 5% CO_2_. Supernatant from each group was harvested at 0, 12 and 24 hpt and clarified by centrifugation at 2500× *g* for 10 min at 4 °C. Clarified supernatants were dispensed into the murine IFN-β and IL-6 as well as human IL-6 and IFN-β capture antibody-coated ELISA plate to measure cytokine secretion. The test was performed in duplicate for IFN-β and human IL-6 and in triplicate for other cytokines. The data shows the representative means ± SD of each murine cytokine measured over time; (**C**) for the determination of Type I IFN-related or NF-κB related protein phosphorylation, cells were harvested at 0, 8, 12 and 24 hpt with LPS or Epimedium koreanum Nakai (EKN) and washed with phosphate-buffered saline (PBS), before being subjected to immunoblot analysis. The samples were separated by SDS-PAGE, transferred onto a PVDF membranes and were probed with the target protein antibodies (anti-IRF3/anti-phopho-IRF3, anti-p65/anti-phopho-p65, anti-STAT1/anti-phopho-STAT1, anti-TBK1/anti-phopho-TBK1, anti-p38/anti-phopho-p38, anti-ERK/anti-phopho-ERK, anti-β-actin) before being visualized with the enhanced chemiluminescence detection system (ECL-GE healthcare) using a ImageQuant LAS 4000 mini (GE healthcare, North Richland Hills, TX, USA).
